# The Effect of Bi on the Kinetics of Growths, Microstructure and Corrosion Resistance of Hot-Dip Galvanized Coatings

**DOI:** 10.3390/ma17225604

**Published:** 2024-11-16

**Authors:** Henryk Kania, Helena Otmačić Ćurković, Jan Kudláček, Angela Kapitanović, Joanna Nackiewicz, Daniel Černý, Grzegorz Konopkin

**Affiliations:** 1 Department of Metallurgy and Recycling, Faculty of Materials Engineering, Silesian University of Technology, Krasińskiego 8, 40-019 Katowice, Poland; 2 Department of Electrochemistry, University of Zagreb Faculty of Chemical Engineering and Technology, Trg Marka Marulića 19, 10000 Zagreb, Croatia; hotmac@fkit.unizg.hr (H.O.Ć.);; 3 Department of Manufacturing Technology, Faculty of Mechanical Engineering, Czech Technical University in Prague, Technická 4, 166-07 Prague, Czech Republic; jan.kudlacek@fs.cvut.cz (J.K.);; 4 Department of Physical Chemistry and Molecular Modeling, Faculty of Chemistry and Pharmacy, University of Opole, ul. Oleska 48, 45-052 Opole, Poland; 5 Faculty of Transport and Aviation Engineering, Silesian University of Technology, Krasińskiego 8, 40-019 Katowice, Poland

**Keywords:** hot-dip galvanizing, zinc baths, corrosion resistance

## Abstract

This paper presents the results of studies on the growth kinetics, microstructure (SEM/EDS) and corrosion behavior of coatings obtained by hot-dip galvanizing process in baths containing Bi additive. The coatings for testing were produced on low-silicon steel in a Zn bath containing 0.04, 0.12 and 0.4 wt.% Bi. The corrosion resistance of the coatings was determined comparatively in standard Neutral Salt Spray Tests (NSST) (ISO 9227) and sulfur dioxide test (SDT) in a humid atmosphere (ISO 22479). Potentiodynamic tests and electrochemical impedance spectroscopy measurements were conducted. It was found that the addition of 0.04 and 0.12 wt.% Bi reduces the total thickness of the coatings and the thickness of intermetallic layers, while the content of 0.4 wt.% Bi in the bath increases the thickness of the layers forming the coating. Direct corrosion tests (NSST and SDT) and electrochemical tests showed that the addition of Bi to the zinc bath reduces the corrosion resistance of the coatings. The corrosion resistance of the coatings decreases with increasing Bi concentration in the zinc bath. In the microstructure of the coatings, it was found that Bi precipitates mainly on the surface of the coating, but also on the cross-section of the outer layer and ζ intermetallic layer. Bi precipitates, due to their cathodic nature, affect the reduction of the corrosion resistance of the coatings with the increase of their content in the bath.

## 1. Introduction

Hot-dip galvanizing coatings are currently widely used as anti-corrosion protection for steel structures. The development of hot-dip technology focuses mainly on obtaining high-quality zinc coatings with controlled thickness and aesthetic appearance, while reducing the cost of its production by reducing zinc consumption. For this purpose, various alloy additives are introduced into the bath, which reduce the tendency to oxidize the bath surface (Al), reduce the reactivity of the steel (Ni) and improve the outflow of liquid zinc from the steel surface.

In the past, the best way to improve the removal of liquid zinc was to add a Pb additive to the bath. Lead lowers the surface tension and improves the fluidity of liquid zinc [1,2]. With a content of 1.2 wt.% Pb, its solubility in liquid zinc reaches a state of saturation [3]. However, zinc baths are characterized by the best properties, including the highest pourability and the lowest surface tension, with a content of 0.4–0.5 wt.% Pb [4]. However, lead is toxic to the environment and human health [5,6], therefore its presence in baths is undesirable. Therefore, for over two decades, lead in baths has been increasingly replaced with the addition of Bi.

Bismuth is not harmful to the environment, and the technological benefits of using the Bi additive have been relatively well verified in industrial practice. However, information regarding the influence of Bi on the properties of the bath as well as the structure and properties of the coating is very limited. Data available in the literature concerns the effects of Bi mainly in multi-component baths, where the synergistic effect of other elements cannot be ignored. The most important information regarding the effect of Bi in zinc baths is provided by Gagne [7,8]. Surface tension has the greatest impact on the drainage of liquid zinc [7]. The results of the sessile drop technique showed that 0.1 wt.% Bi causes a reduction in the surface tension of liquid zinc at a level comparable to the effect of 1.0 wt.% Pb [8]. Schulz [9] shows that the addition of approx. 0.05–0.1 wt.% Bi reduces the surface tension of liquid zinc by approx. 100 mJ/m^2^ (dyn/m^2^), which is comparable to the effect of Pb in the concentration range of 0.4–0.5 wt.%. Gagne [10] also showed in industrial conditions that a bath using Special High Grade (SHG) zinc with the addition of 0.1 wt.% Bi provided similar drainage as observed in a bath using Prime Western Grade (PWG) zinc containing approx. 1 wt.% Pb. Based on this, it is assumed that a 10-fold lower Bi content in the bath will allow for achieving similar effects to those obtained with standard Pb additions to the bath.

Pistofidis et al. [11] studied the effect of Bi on the structure of coatings. They found that Bi is released in the form of inclusions in the outer layer of the coating and at the ζ intermetallic grain boundaries. However, in their research they only compared the structure of coatings obtained in baths containing 1.0 and 2.0 wt.% Bi. The Bi content they tested is in the range of much higher concentrations than those commonly considered optimal. Moreover, the presented coating morphology is characteristic of δ1 intermetallic. Although the authors of [11] confirm the occurrence of ζ intermetallic, the presented EDS analysis is not able to prove the occurrence of ζ intermetallic with sufficient certainty, and the XRD analysis only identifies general peaks for the Fe–Zn phases without distinguishing between the ζ and δ1 phases. In another study, Pistofidis et al. [12] confirm that the coating obtained in a bath containing 1.0 wt.% Bi is composed mainly of δ1 intermetallic. However, the zinc bath additionally contained 1.0 wt.% Ni, so the structure should be explained by the synergistic interaction of these two metals. The results of coating thickness tests [11] indicate that the addition of Bi reduces the coating thickness, but the analysis was carried out only for one value of the immersion time in the bath. The structure of coatings with lower Bi contents in the bath (0.10–0.15 wt.%) was studied by Fratesi et al. [13]. However, their research does not reveal the location of Bi in the coating structure, but only focuses on the synergistic effect of Bi and Ni on limiting the reactivity of steel in the galvanizing bath.

Pistofidis et al. in their publications [11] and [12], draw attention to an important aspect of the impact of the revealed Bi inclusions in the coating structure. They claim that due to the cathodic nature of Bi in relation to Zn, the presence of Bi inclusions may be harmful to the corrosion resistance of coatings. However, their claim was not supported by any research results. This aspect of the impact of Bi, and previously Pb, has not been discussed or sufficiently researched. Gagne [7] reports that atmospheric corrosion tests show no differences in the corrosion resistance of coatings obtained in baths containing Bi. However, studies by authors [14] and [15] of multi-component baths containing Bi show a decrease in corrosion resistance. In these tests, however, the Bi content in the bath is kept constant at approximately 0.05 wt.% and the synergistic effect of other alloy additives such as Al, Ni and Sn cannot be ignored.

The available knowledge about the effect of Bi on the width and structure of the coating is very limited and its application, largely based on industrial experience, ignores a very important aspect of the influence of this alloy addition on the corrosion resistance of coatings. Therefore, this article presents research results determining the effect of Bi addition in a wide concentration range on the growth kinetics, structure and corrosion resistance of coatings. Taking into account its alternative nature in relation to Pb, the tests were conducted on baths with a bismuth content of 0.04 wt.% Bi as a 10-fold lower content than the optimal Pb content, 0.12 wt.% Bi as a 10-fold lower content than the Pb saturation state and 0.4 wt.% Bi as the content corresponding to the optimal Pb content. In these studies, the effect of Bi was separated from the synergistic effect of other alloy additives. The tests will allow determining the Bi content in the bath, leading to the required thickness and structure of the coatings, and predicting their corrosion resistance.

## 2. Experimental

### 2.1. Materials and Hot-Dipping

S235JRG2 steel (ArcelorMittal Poland, Dąbrowa Górnicza, Poland) was used in this research. The chemical composition of the steel was determined using a Spectro Lab M8 emission spectrometer (SPECTRO Analytical Instruments, Kleve, Germany) and is presented in Table 1. The S235JRG2 steel contained 0.21 wt.% Si, which allows it to be classified as a low-silicon steel.

Zinc baths were prepared from high-purity metals: SGH Zinc (99.995%) (ZGH “Bolesław” S.A., Bukowno, Poland) and bismuth (99.99%) (Sigma-Aldrich Chemie GmbH, Steinheim, Germany). In order to eliminate changes in the composition of the baths, pure iron was dissolved in them until saturation was achieved. The chemical composition of the bath was determined using an ARL 3460 emission spectrometer and is presented in Table 1.

Test samples were cut from a 2 mm thick sheet of metal to the following dimensions: 25 × 50 mm^2^ for testing the structure and kinetics of coating growth and to 50 × 100 mm^2^ for corrosion testing. The samples were first degreased in HydronetBase solution for 5 min, pickled in 12% HCl solution for 10 min, rinsed in water and fluxed in DomuFlux60 solution for 2 min. Then, the samples were kept in a forced air dryer for 15 min at 120 °C to remove moisture before immersion in the zinc bath. Then, the samples were immersed in galvanizing baths at a temperature of 450 ± 2 °C for 60, 180, 360 and 720 s. Samples for corrosion tests were immersed for 180 s. After the assumed time, the samples were withdrawn and cooled in air.

### 2.2. Microstructure Characterization

Metallographic examinations were performed using an Olympus GX51 light microscope (Olympus Corporation, Tokyo, Japan). The thickness measurement on the cross-section of the coating layers was carried out using a microscopic method. Each sample was cut at a distance of 20 mm from the upper edge and then a metallographic microsection was performed. This preparation made it possible to unify the location of the coating cross-section and eliminate the influence of the sample geometry on changes in layer thickness. Image registration and layer thickness measurement were performed using analysis software Olysia m3 (Olympus Corporation, Tokyo, Japan). The layer thickness measurement result was the average of 10 randomly selected places on each side of the sample over the entire width of the sample (25 mm).

The examination of the surface microstructure and cross-section of the coatings at high magnifications was performed using Hitachi S-3400 N scanning electron microscopy (SEM) (Hitachi, Tokyo, Japan). The SEM was equipped with an energy dispersive spectroscope (EDS) on which studies of the chemical composition in micro-areas were performed. Microstructure image registration and EDS analysis were performed using Noran Instruments—System Six software (Thermo Fisher Scientific, Waltham, MA, USA).

### 2.3. Corrosion Testing Method

Neutral salt spray tests (NSST) were performed in accordance with the ISO 9227 standard [16]. The tests were carried out in a CORROTHERM Model 610 salt chamber (Erichsen, Hemer, Germany) in the mist of a 5% NaCl solution in distilled water. The temperature in the chamber was 35 ± 1 °C. The exposure time of the samples in the chamber was 24, 48, 96, 240, 480, 720 and 1000 h, respectively.

Sulfur dioxide tests (SDT) in a humid atmosphere were performed in accordance with the ISO 22479 standard [17]. The tests were carried out in a Koesternich Hygrotherm model 519 chamber (Erichsen, Hemer, Germany). The exposure of samples in the chamber was carried out in 24-h cycles under the conditions of Method B: the tested samples were exposed to sulfur dioxide for 8 h and then exposed to standard atmosphere for 16 h. 0.2 dm^3^ of sulfur dioxide was used for each daily cycle. During exposure to sulfur dioxide, the temperature in the chamber was 40 ± 1 °C. The total research time was 30 daily cycles.

After subsequent periods of exposure of the samples in corrosion chambers, unit changes in the mass of the samples were determined according to the equation:

Δ*m* = (*m_t_* − *m_o_*)·*S*^−1^ (1)

where:

*m_o_*—sample mass before testing [g],

*m_t_*—sample mass after exposure time t in the corrosion chamber [g],

*S*—exposure area of specimen [m^2^].

Before measuring the mass, no corrosion products were removed from the surface of the samples. The final result of the mass measurement was the average for five samples of the same type and three measurements for each sample. After completion of NSST, the weight loss of the coatings after removal of corrosion products was additionally determined. Corrosion products were removed in a solution of ammonium chloride (NH_4_Cl) in distilled water (100 g/dm^3^) for 5 min at 70 °C in accordance with the ISO 8407 standard [18].

Electrochemical measurements were conducted in 3.5% NaCl solution, prepared from deionized water and NaCl p.a. (Lachner d.o.o., Zagreb, Croatia). Galvanized steel samples were placed in a flat corrosion cell such that the exposed area was 4.9 cm^2^. They served as working electrodes, while a saturated calomel electrode (SCE) and graphite rod served as the reference and counter electrode, respectively. All potential values in the paper are given relative to the SCE electrode. Measurements were performed using a Bio-Logic SP-300 potentiostat. Before conducting electrochemical studies, samples were left in 3% NaCl solution for one hour, which was sufficient to achieve the stable value of open circuit potential (OCP). Firstly, electrochemical impedance spectroscopy measurements were conducted at OCP with an amplitude of 10 mV and in a frequency range from 100 kHz to 10 mHz. Afterwards, linear polarization measurements were conducted in the potential range ± 250 mV vs. OCP with the scan rate 0.5 mVs^−1^.

## 3. Results and Discussion

### 3.1. Structure and Kinetics Growth of Coatings

**Figure 1:**
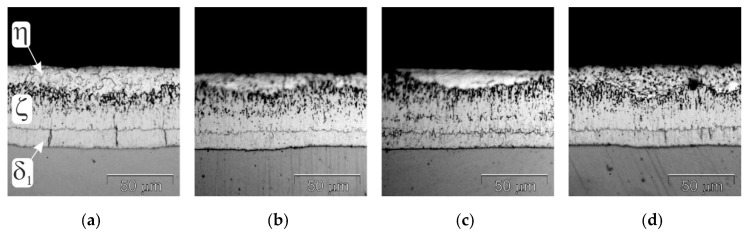
Structure of coatings obtained in: (**a**) Zn, (**b**) Zn-0.04Bi, (**c**) Zn-0.12Bi and (**d**) Zn-0.4Bi. Temperature 450 °C, dipping time 6 min.

The structure of the coatings obtained in the Zn bath and baths with the addition of Bi are shown in Figure 1. The coatings have a very similar layer structure. The morphology of the structural components of the coatings allows us to distinguish the Fe–Zn intermetallic layer composed of δ_1_ intermetallic (FeZn10) and ζ intermetallic (FeZn13), and the outer layer η (solid solution of Fe in Zn). This coating structure is typical for coatings obtained on low-silicon steel and consistent with the literature [9,19].

**Figure 2:**
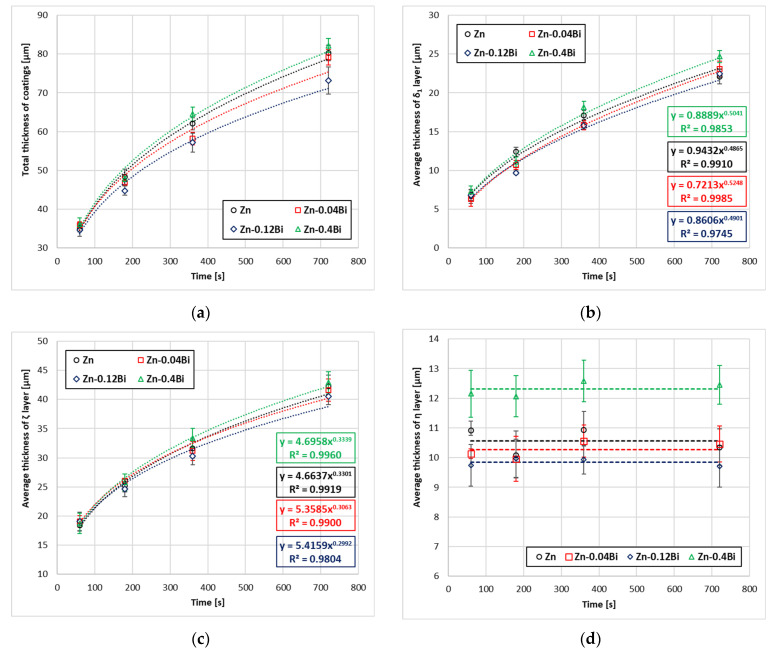
Kinetics of growth of coating: (**a**) total thickness, (**b**) average thickness of δ_1_ intermetallic layer, (**c**) average thickness of ζ intermetallic layer, (**d**) average thickness of outer layer—η.

The dependence of the total thickness of the coating and the thickness of the layers in the coating on the time of immersion in the bath is shown in Figure 2. The tested coatings show a large contribution of the thickness of the layers of intermetallic phases ζ and δ1 in shaping the total thickness of the coating. The total thickness of the coating (Figure 2a) increases in proportion to the thickness of these layers, which also indicates small changes in the thickness of the outer layer η. It can be seen that in the initial stage the increase in the total thickness of the coating is intense, but the rate of thickness increase decreases as the immersion time increases. Content of 0.04 and 0.12 wt.% Bi causes a reduction in the total coating thickness compared to the coating thickness obtained in a Zn bath. After an immersion time of 720 s, coatings with a thickness of 79.14 ± 3.21 µm in the Zn-0.04Bi bath, 73.164 ± 3.53 µm in the Zn-0.12Bi bath and 80.25 ± 1.96 µm in the Zn bath were obtained. However, increasing the additive content to 0.4 wt.% Bi increases the coating thickness to 82.19 ± 1.77 µm, which exceeds the thickness of the coating obtained in a Zn bath.

A similar nature of growth can be observed for the layers of δ1 intermetallic (Figure 2b) and ζ intermetallic (Figure 2c) forming the diffusion layer. The growth kinetics of intermetallic phase layers in the diffusion layer can be described by an exponential relationship:

(2) $$y(thickness)=k\cdot t^{n}$$

where:

y (thickness)—thickness of intermetallic layer [µm],

k—growth rate constant [µm/s],

t—dipping time [s],

n—growth rate time constant.

The determined trend function for the experimental data and the high value of the correlation coefficient R2 confirm the power law nature of the growth of intermetallic layers in the coating (Table 2). The growth rate time constant n is an indicator determining the type of kinetics controlling the growth of the tested layer. The values of the time constant of the growth rate n and the growth rate constant k of the δ_1_ intermetallic layer and ζ intermetallic layer for different contents of Bi in the bath are presented in Table 2. The value of the exponent n describing the growth of the δ_1_ intermetallic layer is close to 0.5, which indicates that the growth of this layer is controlled by diffusion. The values of the n coefficient for the ζ intermetallic layer were smaller and range around 0.3. Values less than 0.5 mean the slower growth of this layer. The growth of the ζ intermetallic layer is therefore slower than the growth of the δ_1_ intermetallic layer. This result can be considered obvious, as many studies indicate similar values of n coefficients in these Fe–Zn intermetallic layers [20]. The slower growth of the ζ intermetallic layer should be explained by the fact that ζ intermetallic is in direct contact with liquid zinc. The diffusive growth of this phase is therefore accompanied by a simultaneous dissolution process at the ζ intermetallic/liquid zinc interface [21], which slows down the final effect of the increase in the thickness of this layer. The growth kinetics show that the addition of 0.04Bi and 0.12Bi causes a decrease in the thickness of both the δ_1_ intermetallic layer (Figure 2b) and the ζ intermetallic layer (Figure 2c). However, a higher content of 0.3Bi causes an increase in thickness above the thickness of those layers obtained in the bath without the addition of zinc. The growth rate time constant n for the ζ intermetallic layer shows that its value for the Zn-0.04Bi (n = 0.3063) and Zn-0.12Bi (n = 0.2992) baths is smaller compared to its value in the Zn bath (n = 0.3301), while it obtains a higher value in the Zn-0.4Bi bath (n = 0.3339). This trend of the dependence of the growth rate time constant n for the ζ intermetallic layer on the Bi content in the bath correlates well with the dependence of its overall thickness on the Bi content. However, these relationships cannot be confirmed for the δ_1_ intermetallic layer. Although the layer thickness of this phase maintains a similar dependence on the Bi content in the bath, the growth rate time constant n for the δ_1_ intermetallic layer has the lowest value for the Zn bath (n = 0.4865). However, the δ_1_ intermetallic layer does not have direct contact with the liquid zinc from which Bi is supplied. The mechanism of Bi influence on the growth of Fe–Zn intermetallic has not yet been clearly explained. Tatarek and Saternus [22], conducting diffusion dissolution studies in stationary conditions, report that in baths containing Bi, the value of the diffusion coefficient D = f(c Fe) decreases in the ranges of occurrence of Fe–Zn intermetallics, and in particular in the range of the occurrence of the ζ phase. This may explain the reduction in the thickness of the ζ intermetallic layer with lower Bi contents in the bath.

The addition of Bi has a similar tendency to influence the thickness of the outer layer of coatings (Figure 2d). In the Zn-0.04Bi and Zn-0.12Bi baths, smaller outer layer thicknesses were obtained than in the Zn bath, while in the Zn-0.4Bi bath its thickness is the largest. However, it should be noted that the thickness of the outer layer η, which is a component of the total thickness, is not subject to the laws of diffusion growth. According to Nernst’s theory, dissolution at the solid–liquid interface takes place with the participation of a stationary layer of liquid directly adjacent to the solid, through which substances diffuse towards the interface [21]. The thickness of the outer layer at a constant temperature and composition of the bath depends largely on the viscosity of the liquid zinc layer saturated with dissolved iron, which is in direct contact with the steel. Therefore, there are difficulties in the practical description of the phenomena shaping the thickness of the outer layer. Nevertheless, it can be seen that the time the sample remains in the bath does not affect the thickness of the outer layer. However, Figure 2d clearly shows the influence of the content of alloy additives in the bath. Content 0.04 wt.% Bi and 0.12 wt.% Bi causes a reduction in the thickness of the outer layer of the coating, while the content of 0.4 wt.% Bi increases its thickness compared to the thickness of the outer layer of the Zn coatings.

### 3.2. Microstructure (SEM) and EDS Analysis of Coatings Surface

**Figure 3:**
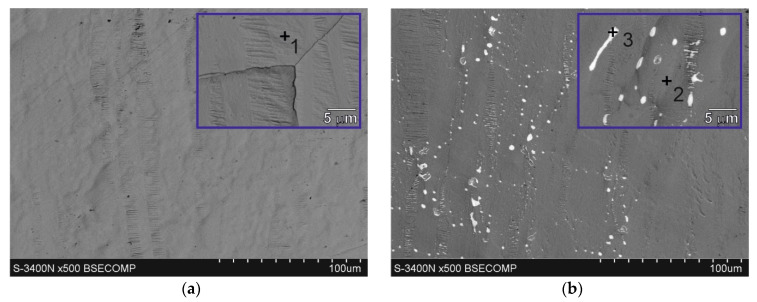

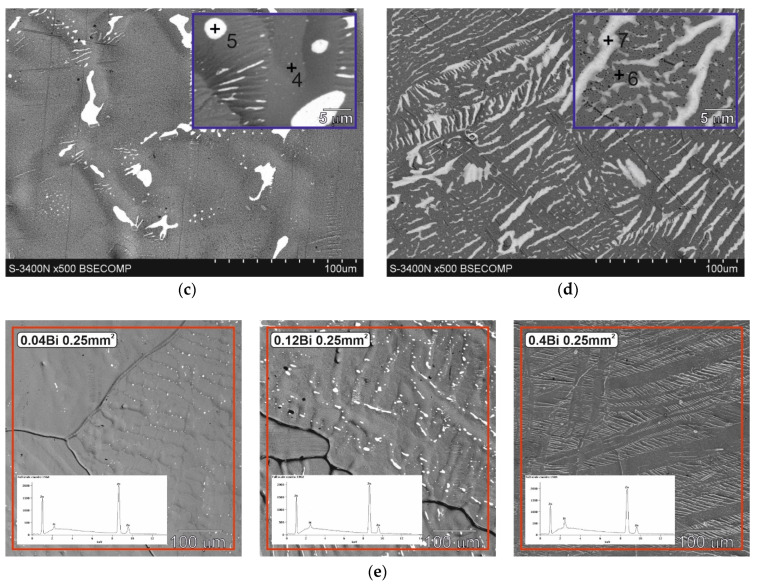
Microstructure (SEM) of coatings surface obained in: (**a**) Zn, (**b**) Zn-0.04Bi, (**c**) Zn-0.12Bi, (**d**) Zn-0.4Bi baths and (**e**) 0.25mm^2^ area with EDS analysis.

The corrosion process is initiated on the coating surface. Therefore, the morphology of the top surface of coatings determines the course of corrosion in its initial stages. Figure 3 shows the microstructure of the coating surface obtained in the Zn bath and in baths with the addition of Bi. The chemical composition in the marked micro-areas is presented in Table 3. The surface of the Zn coating (Figure 3a) shows a homogeneous structure. EDS analysis shows that it is made of “pure” zinc (100 wt.% at point 1), and at high magnifications grain boundaries can be observed. The microstructure and EDS analysis of the surface of coatings obtained in baths containing Bi (Figure 3b–d), regardless of its concentration in the bath, show the occurrence of Bi precipitates (points 3, 5, 7). However, the Bi content in the bath has an intensive influence on the morphology of Bi precipitates. In a bath containing 0.04 wt.% Bi fine Bi precipitates are distributed in the Zn matrix (Figure 3b). An increase in the Bi content in the bath to 0.12 wt.% Bi changes the morphology of the precipitates. Bi precipitates are much larger in size, but fine banded Bi precipitates can also be observed in the structure (Figure 3c). However, the morphology of the precipitates on the surface of the coating obtained in the bath containing 0.4 wt.% Bi changes significantly. The precipitates take a band shape and have a clearly directed structure (Figure 3d). Bi–Zn phase diagrams both presented by Massalski [23] and Malakhov [24] show that in the solid state both bismuth is insoluble in zinc and zinc is insoluble in bismuth. On this basis, it can be concluded that Bi precipitates are released from the solid zinc solution during the crystallization of the outer layer of the coating, and the Zn content in the analysis of the chemical composition of the precipitates results from the possibility of detecting the elements using the EDS method. EDS analysis in the Zn matrix of coatings obtained in the Zn-0.12Bi bath (point 4) and in the Zn-0.4Bi bath (point 6) shows the content of small amounts of Bi (0.2 wt.%). Due to the lack of solubility of Bi in Zn in the solid state, this may indicate that Bi also forms finely dispersed precipitates in the zinc matrix at higher Bi contents in the bath. EDS microanalysis of the chemical composition performed on the coating surface in an area of 0.25 mm2 (Figure 3e) showed the occurrence of 0.7 wt.% Bi in the Zn-0.04Bi bath, 1.1 wt.% Bi in the Zn-0.12Bi bath and 3.6 wt.% Bi in the Zn-0.4Bi bath. This shows that on the one hand the Bi content on the coating surface does not change in proportion to the Bi content in the bath, but on the other hand this Bi content on the bath surface is much higher than the Bi content in the bath. The microstructure from the larger surface of the coating (Figure 3e) also shows their location mainly in the interdendritic recesses of the zinc matrix, and also their directed distribution at higher Bi contents.

### 3.3. Cross-Section Microstructure of Coatings

**Figure 4:**
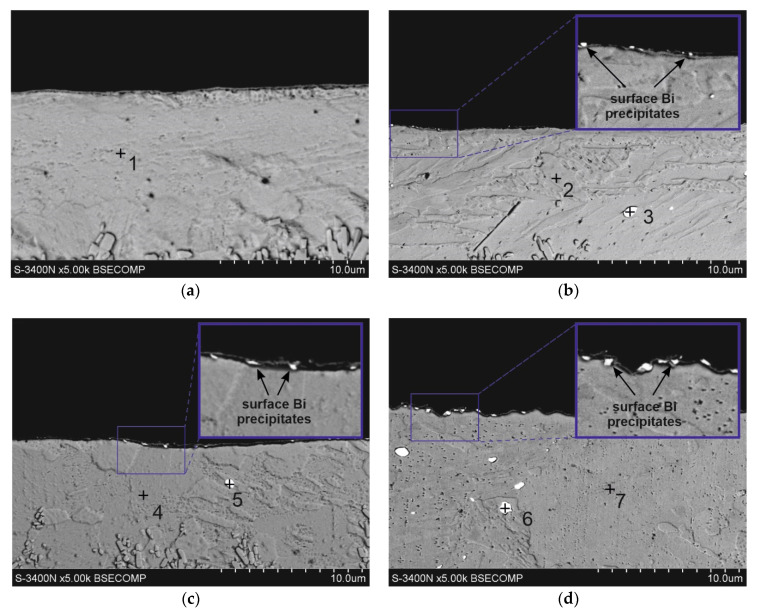
Microstructure (SEM) of outer layer of coating obtained in: (**a**) Zn, (**b**) Zn-0.04Bi, (**c**) Zn-0.12Bi and (**d**) Zn-0.4Bi baths.

Investigations of the microstructure of the coatings showed that Bi is also released on the cross-section of the coating. Figure 4 shows the microstructure of the outer layer of coatings obtained in the baths with the addition of Bi, along with marked areas of the EDS analysis, the results of which are summarized in Table 4. The outer layer is formed by a solid solution η, in which the EDS analysis confirms the occurrence of 100 wt.% Zn (points 1, 2, 4, 7). Bi is precipitated in the form of precipitates with a regular, almost spherical shape (points 3, 5, 6). The size of these precipitates is much larger than Bi surface precipitates (Figure 4 b–d). However, they are randomly distributed in the solution matrix η as single precipitates, although in the case of a content of 0.4 wt.% Bi, clusters of several Bi precipitates of different sizes may form in the bath (Figure 4d).

**Figure 5:**
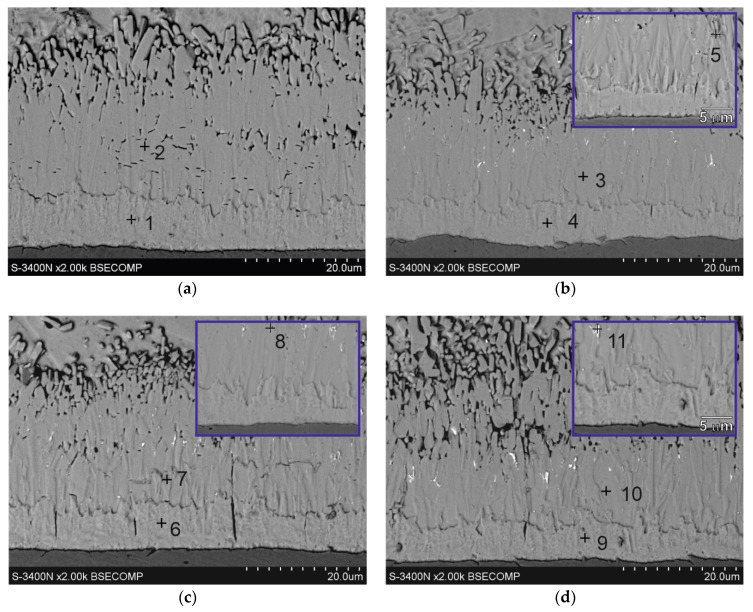
Microstructure (SEM) of diffusion layer of coating obtained in: (**a**) Zn, (**b**) Zn-0.05Bi, (**c**) Zn-0.1Bi and (**d**) Zn-0.3Bi baths.

SEM images and EDS analysis of the cross-section of the coating show that Bi is also released in the diffusion layer of the coating (Figure 5, Table 5). According to the Fe–Zn equilibrium system [23], the content of Fe and Zn in the range of 6.2–6.5 wt.% for Fe and 94.1–95.3 wt.% for Zn (in points 2, 3, 7, 10), respectively, is in very good agreement with the content of Fe and Zn in ζ intermetallics, while the content of 7–11.5 wt.% for Fe and 93–88.5 wt.% for Zn (in points 1, 4, 6, 9) is within the homogeneity range of δ1 intermetallics. No Bi content was found in the composition of these phases. This may indicate the lack of Bi solubility in the phases of the Fe–Zn system. The lack of Bi solubility has not been confirmed in the literature, but it seems very likely. In the ζ intermetallic layer, bright precipitates containing Bi are visible (points 3, 7, 10). The morphology of the ζ intermetallic layer shows that the privileged place for crystallization of Bi precipitates is at the crystal boundaries. However, the morphology of the δ1 intermetallic layer is more compact, hence, the location of Bi precipitates in it may be impossible. As growth kinetics studies show, the growth of Fe–Zn intermetallic layers occurs with a smaller (ζ intermetallic—Figure 2c) or larger (δ1 intermetallic—Figure 2b) participation in the diffusion process. As a result of the reaction diffusion of Fe and Zn, Fe–Zn intermetallic phases are formed [25]. The lack of Bi in the composition of these phases may indicate that Bi is not involved in the growth of both the ζ intermetallic and δ1 intermetallic layers. However, the ζ phase dissolves in contact with liquid zinc and then crystallizes again from the iron-saturated zinc solution [26]. Due to the lack of solubility, bismuth remains in the zinc solution, but due to the crystalline structure of the ζ intermetallic layer, some Bi can be trapped in the spaces between the ζ intermetallic crystals during its secondary crystallization. This may explain the presence of Bi precipitates in the ζ intermetallic layer, but for the unequivocal acceptance of this hypothesis, it is necessary to experimentally verify the knowledge about the solubility of Bi in the intermetallic phases of the Fe–Zn system.

Assuming the lack of Bi solubility in ζ and δ1 intermetallics and the high solubility of Bi in liquid zinc [23,24], it should be assumed that Bi is distributed in the outer layer during its crystallization. Additionally, the consumption of zinc for the formation of Fe–Zn intermetallics may increase the Bi concentration in the layer of liquid zinc directly adjacent to the ζ intermetallic layer, which is responsible for the formation of the outer layer of coating. However, studies of the microstructure of the coatings showed a greater tendency for Bi to be distributed on the coating surface than to be located on the cross-section of the coating. Previous research conducted by Kopyciński [27] has shown that during the crystallization of the outer layer of coatings, heterogeneous nucleation of zinc crystals occurs at the ζ intermetallic/liquid zinc boundary. This leads to the growth of zinc dendrites towards the surface of the outer layer. This growth of dendrites occurs even though there should be a lower temperature on the surface of the still liquid zinc layer. However, in the interdendritic spaces, heat flow towards the coating surface is still possible, and the nucleation and growth of dendrites is possible due to subcooling in this area. Based on the mechanism presented by Kopyciński [27], it can be assumed that the crystallization front of the outer layer moves from the surface of the ζ intermetallic layer to the surface of the outer layer. At the same time, the crystallization of liquid zinc takes place at a temperature of 419.5 °C, while Bi melts at a temperature of 271.4 °C [23,24]. The lack of solubility of Bi in the solid state and the high solubility of Bi in liquid zinc will lead to Bi being located on the crystallization front and ultimately on the coating surface. This may explain the high concentration of Bi precipitates on the coating surface, especially with the high Bi content in the bath.

### 3.4. Results of Corrosion Resistance

#### 3.4.1. Neutral Salt Spray Test (NSST)

**Figure 6:**
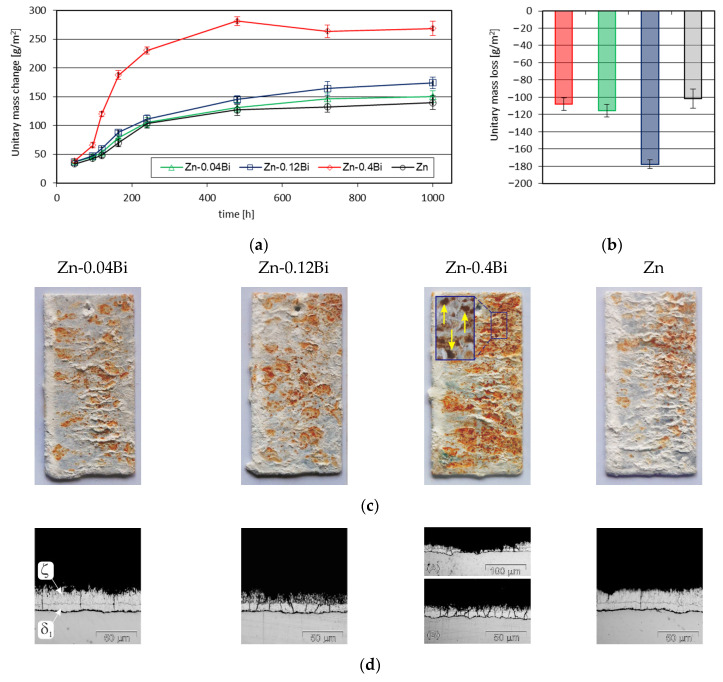
Results of corrosion resistance tests in NSST: (**a**) unit mass change of coatings during exposure in a salt spray chamber, (**b**) mass loss of coatings after completion of NSST, (**c**) appearance of coatings after completion of NSST and (**d**) structure of the cross-section of coatings after completion of NSST.

Figure 6 shows the results of corrosion resistance tests of coatings in NSST. The dependencies of unit mass changes of the coatings (Figure 6a) during exposure in the salt chamber show that all coatings were characterized by an increase in mass. The greatest increase in the mass of corrosion products was observed in the coating obtained in the bath with the addition of 0.4 wt.% Bi. After completing the corrosion test (1000 h), the unit weight increase of this coating was 268.36 ± 12.26 g/m^2^. The smallest weight increase was observed for the coating obtained in a Zn bath without the addition of Bi—139.57 ± 11.86 g/m^2^. For coatings obtained in a bath with the addition of 0.04 wt.% and 0.12 wt.% Bi, the weight gain was 150.32 ± 10.01 g/m^2^ and 174.17 ± 9.88 g/m^2^, respectively. Increasing the Bi content in zinc bath causes the formation of more corrosion products on the surface of the coatings. The final increase in the mass of corrosion products shows that in the Zn-0.4Bi bath, the amount of coating corrosion products is approximately 1.5 times greater than in the Zn-0.12Bi bath and almost 2 times greater than in the Zn bath. At the same time, the mass increases of corrosion products of coatings formed in Zn-0.04Bi and Zn-0.12Bi baths are only slightly higher compared to the Zn bath. This is also confirmed by the weight loss of the coatings after removing corrosion products from the coating surfaces (Figure 6b). After completion of NSST, the mass of removed corrosion products was 177.54 ± 5.27 g/m^2^ in the Zn-0.4Bi bath, 115.39 ± 7.25 g/m^2^ in the Zn-0.12Bi bath and 108.03 ± 7.44 g/m^2^ in the Zn-0.04Bi bath, respectively. In the Zn bath, it reached the value of 101.57 ± 10.90 g/m^2^.

Analyzing the course of the kinetics of the mass increase of corrosion products on the surface of the coatings (Figure 6a), it can be clearly seen that in the initial phase of the corrosion process, the mass increase of the corrosion products is very rapid. A particularly large increase in mass is observed on the coating obtained in the Zn-0.4Bi bath. During 240 h of NSST, the weight gain of this coating was 230.15 ± 8.04 g/m^2^. This represents 85% of the final weight gain of the corrosion products on this coating. In the initial phase of the corrosion process, the outer layer of the coating performs protective functions. This result shows that corrosion is most intense in the outer layer. At the same time, the coating is covered with white corrosion products, which are identified as zinc corrosion products [28]. However, after completing the corrosion test, red corrosion products can also be observed on the coating surface (Figure 6c), which are identified as iron corrosion products. This proves that the corrosion process takes place in the diffusion layer of the coating. This corrosion course is typical for Fe–Zn intermetallics rich in iron [29]. The appearance of the coatings in Figure 6c clearly shows that the share of red corrosion products increases with increasing Bi content in the bath. This is confirmed by the coating structure in the cross section (Figure 6d). After completion of NSST, the degradation of the coating occurs mainly in the ζ intermetallic layer. However, in baths containing Bi, greater wear of the ζ intermetallic layer can be observed, and with a content of 0.4 wt.% Bi, the corrosion wear reaches up to the δ1 intermetallic layer. In the Zn-0.4Bi bath, local breakthroughs of the coating into the substrate were also observed (Figure 6c—yellow arrow). Local penetration of the coating into the substrate is also confirmed by the cross-sectional image of the microstructure visible in Figure 6d (A).

#### 3.4.2. Sulfur Dioxide Test (SDT) in a Humid Atmosphere

**Figure 7:**
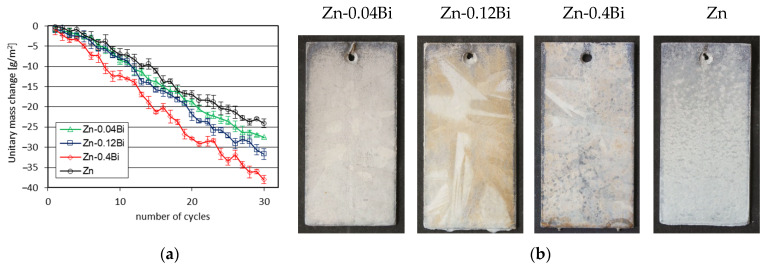
Results of corrosion resistance tests in SDT: (**a**) unit mass change of coatings during exposure in the Koesternich chamber and (**b**) appearance of coatings after the corrosion test.

Figure 7 shows the results of testing the corrosion resistance of coatings in SDT. Changes in the mass of the coatings during exposure in the Koesternich chamber (Figure 7a) show that regardless of the content of Bi additives in the bath, the coatings are characterized by a continuous loss of mass. This means that corrosion products formed on the surface are constantly dissolved in the test environment. After completing the corrosion test in the Koesternich chamber, the weight loss of the coatings increased with the increase in the Bi content in the bath and reached a value from 27.46 ± 0.32 g/m^2^ in the Zn-0.04Bi bath to 38.02 ± 0.99 g/m^2^ in the Zn-0.4Bi bath. The smallest weight loss was observed in the coating obtained in a bath without the addition of Bi. After 30 test cycles, the weight loss of the coating obtained in the Zn bath was 24.08 ± 1.04 g/m^2^.

After completing the corrosion test, the tested coatings did not show any penetration into the substrate. Their appearance was gray and dull (Figure 7b). At the same time, the occurrence of spangles can be observed on the surface of coatings obtained in baths containing Bi. This proves that the corrosion process took place in the outer layer of the coating.

#### 3.4.3. Corrosion Resistance Determined via an Electrochemical Test

**Figure 8:**
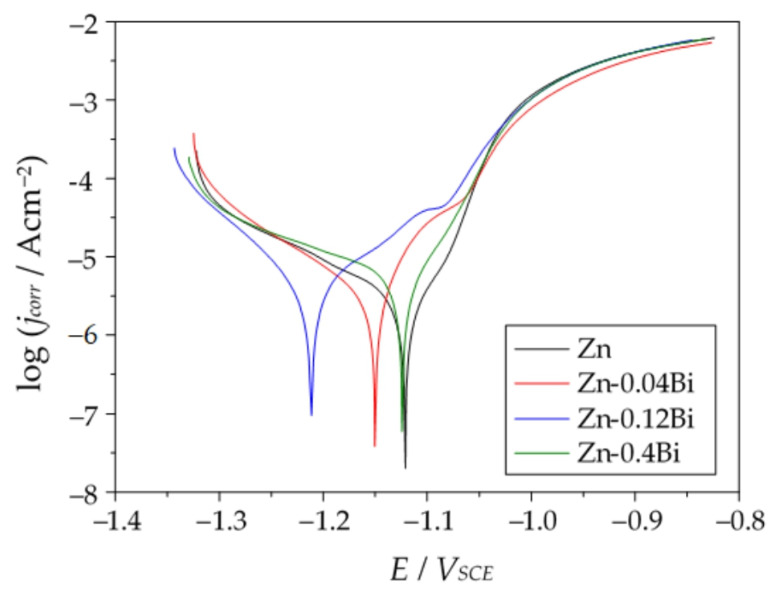
Polarization curves of galvanized steel in 3.5% NaCl solution.

Electrochemical measurements were conducted in 3.5%NaCl solution after one hour of immersion. In this way, the initial corrosion resistance of studied galvanized steel samples was examined. Representative polarization curves are presented in Figure 8. Polarization curves for samples obtained in baths with different Bi content do not exhibit significant difference although it is visible that the sample from the Zn bath shows the lowest current densities in the region near corrosion potential. The Tafel extrapolation method was used to determine corrosion current densities (j_corr_) and corrosion potentials (E_corr_) for all studied samples (Table 6). The obtained corrosion parameters show that corrosion potential values are very similar for all samples, but corrosion current densities are higher for samples from Bi containing baths. They increase in the following order: Zn < Zn-0.04Bi < Zn-0.12Bi. Besides the increase in j_corr_ values, an increase in standard deviation values was observed, in the same order. For Zn-0.4Bi, parameters in Table 6 are calculated from only part of the measurements as 1/3 samples exhibited much higher corrosion current densities (100 times higher) due to the increase of cathodic current densities. This phenomenon shows that the presence of Bi precipitates can result in increased corrosion of Zn coating, but the effect probably depends on the size and distribution of Bi precipitates in the coating.

**Figure 9:**
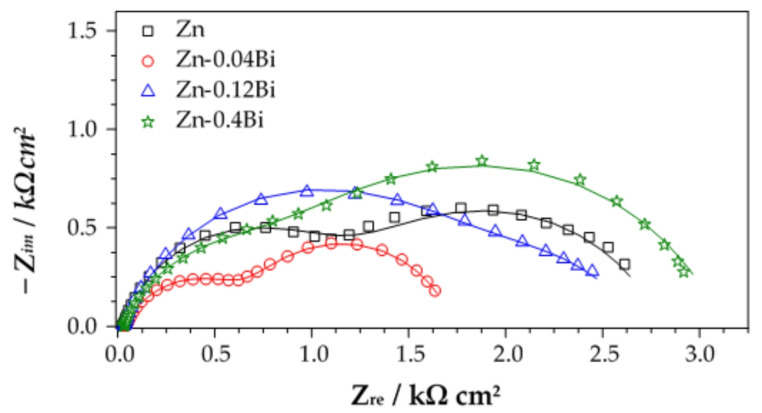
EIS spectra for galvanized steel samples in 3.5% NaCl solution. Symbols present experimental and lines fitted data.

**Figure 10:**
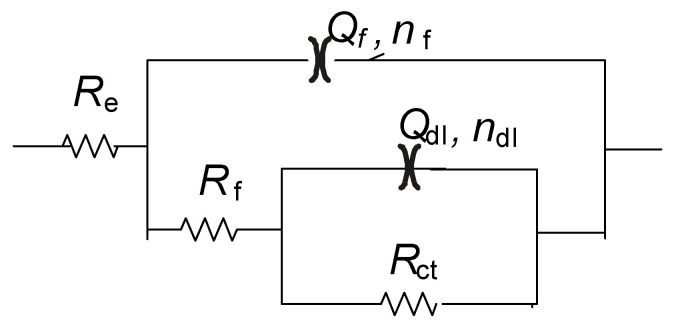
Selected equivalent electrical circuit for analysis of EIS spectra.

In addition to polarization measurements, electrochemical impedance spectroscopy measurements were conducted. Representative impedance spectra of samples prepared in different baths are shown in Figure 9. All spectra exhibit two semicircles, more or less separated. For that reason, the spectra were analyzed with the equivalent electrical circuit consisting of two R–Q pairs (Figure 10), where R is resistance and Q is a constant phase element describing non-ideal capacitive behavior. The R_f_–Q_f_ pair describes the properties of an oxide (corrosion products) layer formed on the top of Zn coating, while R_ct_–Q_dl_ correspond to charge transfer resistance and electrochemical double layer capacitance. R_el_ represents electrolyte resistance between the reference and the working electrode.

Impedance parameters obtained by fitting EIS spectra are presented in Table 7. By comparing the parameters of samples from the Zn and Zn-0.04Bi baths it is clearly observed that both oxide film resistance and charge transfer resistance decrease due to the presence of Bi in the coating. With further increase in Bi content (Zn-0.12Bi), charge transfer resistance decreased even more which indicates increased dissolution of the coating. On the other hand, film resistance, R_f_, increased which can be attributed to the accumulation of corrosion product on the coating surface. For the sample with the highest Bi content, some increase in resistance values is observed. However, it should be stressed that for samples from Zn-0.12Bi and Zn-0.4Bi baths the dispersion of results was higher than for the samples from the other two baths, which confirms previous findings on variation of surface properties on such prepared samples.

The presented corrosion tests show that the addition of Bi has an adverse effect on the corrosion resistance of coatings. This confirms the thesis put forward by Pistofidis et al. [11], who claim that the presence of Bi precipitates in the coating structure favors the formation of corrosion cells. Bi has a positive standard potential, (E°(Bi^3+^/Bi) = 0.308 V, E°(Bi^+^/Bi) = 0.5 V, vs. SHE [30]). Therefore, in contact with the zinc matrix (E°(Zn^2+^/Zn) = −0.7618 V vs. SHE [30]), Bi precipitates will be the cathode. The large potential difference determines that the corrosion cell created by these metals is much more effective than the cell that creates zinc with the release of Pb (E°(Pb^2+^/Pb) = −0.1262 V vs. SHE [30]). This is confirmed by the research of Radu and Vlad [31], in which the coating obtained in a bath containing 0.71 wt.% Bi is characterized by a higher value of corrosion current compared to the coating obtained in a bath containing 0.72 wt.% Pb. However, although the content of Bi and Pb was comparable, the test bath contained Ni and Sn additives. Therefore, a synergistic effect of all alloying additives cannot be ruled out in this case. In another study, Radu et al. [32] show that coatings obtained in a bath containing 0.4 wt.% Bi have lower corrosion current densities. However, they explain the increase in corrosion resistance by the increase in the Al content in the bath in the range from 0.1 wt.% to 0.3 wt.%. The authors do not explain the reasons for the increase in corrosion resistance. However, a high Al content in the bath tends to create a passive Al_2_O_3_ layer on the coating surface. The synergistic effect of Al in the bath does not allow for a clear assessment of the influence of Bi on the corrosion resistance of the coatings. Additionally, the impact of other alloy additives can effectively change the picture of the corrosive impact of the coating, and at the same time, electrochemical tests are not able to simulate the long-term corrosion behavior of coatings.

The tests carried out allow for the clear elimination of the influence of other alloy additives in the bath. Direct comparative corrosion tests supplemented with electrochemical tests show a reduction in the corrosion resistance of the coatings, especially in the initial stage of corrosion. During this time, the outer layer of the coating, in which Bi precipitates are located, undergoes corrosion. In particular, their presence on the coating surface can be an effective corrosion factor initiating and accelerating the degradation of the coating. However, their presence in the diffusion layer of the coating can effectively accelerate corrosion also in the ζ intermetallic layer. The correlation of the structure of the coatings and the results of corrosion tests shows that increasing the Bi content in the bath increases the amount of Bi precipitates on the coating surface, and at the same time, a decrease in the corrosion resistance of the coatings was demonstrated in all corrosion tests performed. This is especially visible at the content of 0.4 wt.% Bi. At the same time, studies on the kinetics of coating growth show that such a high Bi content leads to the formation of thicker coatings.

## 4. Conclusions

The effect of Bi addition to galvanizing baths on the growth kinetics, microstructure and corrosion resistance of the coatings was investigated. Based on the research conducted, the following conclusions were formulated:

- The addition of 0.04 and 0.12 wt.% Bi reduces the total thickness of the coatings and their intermediate layers, including the outer layer η, the ζ intermetallic layer and the δ1 intermetallic layer. An increase in the Bi content in the bath to 0.4 wt.% increases the thickness of the total coating and the thickness of its intermediate layers;
- In the microstructure of the coatings obtained in baths containing Bi, the release of Bi was found mainly on the coating surface, but also on the cross-section of the outer layer and ζ intermetallic layer. The amount of Bi precipitates increases with the increase in the Bi content in the bath;
- Direct corrosion tests (NSST and SDT) showed an increase in corrosion wear in the initial stage of the corrosion process and its relationship with the increase in Bi content in the bath;
- Electrochemical tests show that the addition of Bi results in lower corrosion resistance of the coating. For higher Bi concentrations, an increase in the dispersion of corrosion parameters is observed;
- The presence of Bi precipitates on the coating surface due to the positive standard potential may initiate and accelerate the outer layer η corrosion process, but also accelerate the ζ intermetallic layer corrosion process.

## Figures and Tables

**Table 1:** Chemical composition of S235JRG2 steel and of research baths.

		Content [wt.%]					
		C	Si	Mn	S	P	Fe and others
Steel	S235JRG2	0.137	0.021	0.637	0.004	0.006	rest
		**Al**	**Fe**	**Pb**	**Bi**	**Sn**	**Zn and others**
Bath	Zn	0.0004	0.034	0.001	0.001	0.0001	rest
	Zn-0.04Bi	0.0005	0.031	0.001	0.044	0.0001	rest
	Zn-0.12Bi	0.0004	0.033	0.001	0.11	0.0001	rest
	Zn-0.4Bi	0.0004	0.033	0.001	0.36	0.0001	rest

**Table 2:** Trend function equation of growth kinetics and value growth rate time constant *n* for δ_1_ and ζ intermetallic layers.

Phase	Bath	Trend Function Equation	Correlation Coefficient R^2^	Growth Rate Constant *k* [µm/s^n^]	Growth Rate Time Constant *n*
ζ intermetallic	Zn	y = 4.6637x^0.3301^	0.9919	4.6637	0.3301
	Zn-0.04Bi	y = 5.3585x^0.3063^	0.9900	5.3585	0.3063
	Zn-0.12Bi	y = 5.4159x^0.2992^	0.9804	5.4159	0.2992
	Zn-0.4Bi	y = 4.6958x^0.3339^	0.9960	4.6958	0.3339
δ_1_ intermetallic	Zn	y = 0.9432x^0.4865^	0.9910	0.9432	0.4865
	Zn-0.04Bi	y = 0.7213x^0.5248^	0.9985	0.7213	0.5248
	Zn-0.12Bi	y = 0.8606x^0.4901^	0.9745	0.8606	0.4901
	Zn-0.4Bi	y = 0.8889x^0.5041^	0.9852	0.8889	0.5041

**Table 3:** Results of EDS analysis of the top surface of coatings (analysis points as shown in Figure 3).

Point No.	Zn		Bi	
	wt.%	at.%	wt.%	at.%
1	100.0	100.0	-	-
2	100.0	100.0	-	-
3	38.7	66.9	61.3	33.1
4	99.8	99.9	0.2	0.1
5	22.8	48.5	77.2	51.5
6	99.8	99.9	0.2	0.1
7	21.1	46.1	78.9	53.9
**Area 0.25 mm^2^**				
0.04 Bi	99.3	99.8	0.7	0.22
0.12 Bi	98.9	99.7	1.1	0.35
0.4 Bi	96.4	98.8	3.6	1.16

**Table 4:** Results of EDS analysis of the top surface of coatings (analysis points as shown in Figure 4).

Point No.	Zn		Bi	
	wt.%	at.%	wt.%	at.%
1	100.0	100.0	-	-
2	100.0	100.0	-	-
3	22.9	48.7	77.1	51.3
4	100.0	100.0	-	-
5	26.1	53.0	73.9	47.0
6	11.7	29.7	88.3	70.3
7	100.0	100.0	-	-

**Table 6:** Corrosion parameters determined from polarization curves presented in Figure 8.

Type of Coatings	*j_corr_* μAcm^−2^	*E_corr_* mV_SCE_
**Zn**	2.34 ± 0.08	−1120 ± 17
**Zn-0.04Bi**	3.21 ± 0.36	−1130 ± 21
**Zn-0.12Bi**	4.13 ± 2.23	−1115 ± 58
**Zn-0.4Bi**	2.94 ± 0.36	−1121 ± 30

**Table 7:** Impedance parameters determined from EIS spectra presented in Figure 9.

Type of Coatings	*Q*_f_ µFcm^−2^	*n*_f_	*R*_f_ Ωcm^2^	*Q*_dl_ µFcm^−2^	*n*_dl_	*R*_ct_ Ωcm^2^
**Zn**	48	0.82	1260	969	0.74	1513
**Zn-0.04Bi**	105	0.74	708	1594	0.82	980
**Zn-0.12Bi**	123	0.78	1896	3342	0.67	754
**Zn-0.4Bi**	78	0.77	1125	472	0.74	1957

**Table 5:** Results of EDS analysis of the cross-section of coatings (analysis points as shown in Figure 5).

Point No.	Fe		Zn		Bi	
	wt.%	at.%	wt.%	at.%	wt.%	at.%
1	6.3	7.3	93.7	92.7		
2	9.7	11.2	90.3	88.8		
3	8.8	10.2	91.2	89.8		
4	6.1	7.1	93.9	92.9		
5	5.2	6.3	88.6	91.7	6.2	2.0
6	6.9	8.0	93.1	92.0		
7	10.2	11.7	89.8	88.3		
8	6.1	7.5	84.8	89.5	9.1	3.0
9	5.5	6.4	94.5	93.62		
10	8.4	9.7	91.6	90.30		
11	4.9	6.2	82.8	89.62	12.3	4.2

## Data Availability

Data is contained within the article.
